# Temperature-Corrected Fluidic Glucose Sensor Based on Microwave Resonator

**DOI:** 10.3390/s18113850

**Published:** 2018-11-09

**Authors:** Chorom Jang, Jin-Kwan Park, Hee-Jo Lee, Gi-Ho Yun, Jong-Gwan Yook

**Affiliations:** 1 Department of Electrical and Electronic Engineering, Yonsei University, Seoul 03722, Korea; chorom@yonsei.ac.kr (C.J.); paladin91@yonsei.ac.kr (J.-K.P.); 2 Department of Physics Education, College of Education, Daegu University, Gyeongsan 38453, Korea; hjlee@daegu.ac.kr; 3 Department of Information and Communications Engineering, Sungkyul University, Gyeonggi-Do 14097, Korea; ghyun@sungkyul.ac.kr

**Keywords:** fluidic glucose sensor, complementary split-ring resonator, non-invasive detection, microwave, temperature correction, electromagnetic biosensor

## Abstract

In this paper, a fluidic glucose sensor that is based on a complementary split-ring resonator (CSRR) is proposed for the microwave frequency region. The detection of glucose with different concentrations from 0 mg/dL to 400 mg/dL in a non-invasive manner is possible by introducing a fluidic system. The glucose concentration can be continuously monitored by tracking the transmission coefficient $S_{21}$ as a sensing parameter. The variation tendency in $S_{21}$ by the glucose concentration is analyzed with equivalent circuit model. In addition, to eradicate the systematic error due to temperature variation, the sensor is tested in two temperature conditions: the constant temperature condition and the time-dependent varying temperature condition. For the varying temperature condition, the temperature correction function was derived between the temperature and the variation in $S_{21}$ for DI water. By applying the fitting function to glucose solution, the subsidiary results due to temperature can be completely eliminated. As a result, the $S_{21}$ varies by 0.03 dB as the glucose concentration increases from 0 mg/dL to 400 mg/dL.

## 1. Introduction

Diabetes mellitus, which is a disease caused by insulin malfunction, is a crucial worldwide health issue because of a rapid increase in the number of patients and the lack of treatment. According to the International Diabetes Federation (IDF) report, the expected number of diabetic patients by 2030 was 439 million in 2010 [1], but 552 million in 2011 [2]. This demonstrates that the number of diabetics increases at an unpredictable rate every year. Because the diabetics cannot physiologically control their blood glucose levels within the normal range, they have to check their blood glucose levels frequently to take appropriate action when a sudden change in glucose level is detected. In the case of hyperglycemia, which is defined as a blood glucose level that exceeds 230 mg/dL [3], is a severe status because maintaining high blood glucose level can cause further complications such as renal failure, stroke, cardiovascular disease, eye disease, and foot problems [4,5]. To treat the hyperglycemia, the patients have to inject insulin under the skin. Another case of abnormality in the blood glucose level, hypoglycemia, is defined as a glucose level less than 65 mg/dL and is more fatal than hyperglycemia due to a higher rate of mortality and brain damage [6]. When the hypoglycemia symptom occurs, the patients have to eat sweets until their blood glucose levels are within the normal range. Therefore, continuous monitoring is essential for the diabetics to reduce the danger of additional diseases.

The majority of commercial glucose sensors can detect accurate blood glucose levels. However, patients must prick their fingertips with a lancet to take a blood sample [7]. This process is fearful and painful because patients have to check their blood glucose levels several times a day. To solve this problem, minimally invasive approaches have gained considerable interest in the last few decades [8,9]. Although these methods can reduce the pain from pricking the fingertip, their economic cost remains a crucial issue due to the disposable lancet and strip that are used for detection. In addition, the risk of infection is another concern because pricking the skin may cause scarring even in very small areas [10]. For these reasons, a non-invasive method is good news to the diabetics. Numerous studies have been performed to detect the glucose concentration level based on non-invasive methods. Reverse iontophoresis [11], which is a method of measuring the glucose level by placing an anode and cathode on the skin surface and then applying an electric potential between two electrodes, is a method of non-invasive detection. However, reverse iontophoresis causes skin irritation because it requires an electric potential to be applied to the skin. Bioimpedance spectroscopy can also be a candidate for non-invasive detection. This method can be employed to detect the blood glucose concentration through the impedance spectrum based on the membrane potential change in red blood cells [12]. However, this technique is influenced by the water content and disease that influences the cell membrane potential. Another non-invasive technique is photoacoustic spectroscopy, which is based on the interaction between the incident laser beam of a particular wavelength and the tissue cells that cause changes in heat and pressure [13]. This method is not affected by water, unlike bioimpedance spectroscopy; however, it is vulnerable to pressure, temperature and other environmental factors. Considering these limitations, a microwave sensing technique can be a suitable candidate of the non-invasive approach due to the penetrating ability of the electromagnetic wave. A microwave resonator, a kind of microwave devices, is easy to fabricate, cost-effective, reusable, portable and has low power consumption [14]. In addition, their physical size can be reduced by designing to operate at a higher frequency range. Due to these advantages, in recent decades, microwave resonators have been employed as sensing devices, such as gas sensors [15], temperature sensors [16], pH sensors [17], and in biomolecule detection [18,19], vital sign detection [20,21], wrist pulse monitoring [22], breast tumor detection [23], humidity sensors [24], and so on. In addition, many studies have been proposed to detect the glucose level using a microwave technique [25,26,27,28], but they employed an invasive approach in which the sensor and glucose solution are in direct contact.

Sensors must exhibit stable performance in any environments. In order to ensure the reliable performance of the sensor, it is essential to eliminate additional factors, excluding analytes, that affect the sensing parameter used for detection. If the additional factor cannot be removed, such as an ambient temperature, the sensor must calibrate its effect to achieve a valid performance. The ambient temperature is a significant factor in the electrical properties such as dielectric constant and loss tangent of the liquids [29]. It is indicated that the ambient temperature causes serious errors in the detection of the glucose level [30]. Kim et al. in [31] proved that the detection errors are more sensitive to ambient temperature than humidity. These mean that the sensors detecting the liquid analytes from the difference in electrical properties can be degraded performance by the ambient temperature. Therefore, in the case of the liquid analytes, the temperature factor must be taken into account.

In this study, a glucose sensor that is based on a complementary split-ring resonator (CSRR) is proposed for non-invasive and continuous monitoring of glucose concentrations in the microwave regime with a fluidic system. Additionally, the temperature correction is performed to the detection so that the performance is reasonable.

## 2. Materials and Methods

### 2.1. Material Property Specification

The samples under test (SUT) are prepared with four different glucose concentrations from 0 mg/dL to 400 mg/dL with a step of 100 mg/dL, considering the possible range of human blood glucose levels (from 30 mg/dL to 400 mg/dL) including the concentration of hypoglycemia and hyperglycemia states. Prior to designing the sensor, it is notable that the microwave sensing technique uses the difference in the electrical properties of the SUT for detection. The electrical properties of the SUT are measured using the vector network analyzer (Agilent Technologies, E8364A, Santa Clara, CA, USA) with an open-ended dielectric probe and analysis software (85070D) that derives the complex relative permittivity from reflection coefficient. The complex relative permittivity is defined as

(1) $$\begin{array}{c} \varepsilon_{c}=\varepsilon^{\prime }-j\varepsilon^{\prime \prime }. \end{array}$$

The loss tangent (tanδ), another parameter representing the characteristics of the material, is defined as the ratio between real and imaginary parts of complex relative permittivity, i.e.,

(2) $$\begin{array}{cc} \tan\delta = & \frac{\varepsilon^{\prime \prime }}{\varepsilon^{\prime }}. \end{array}$$

**Figure 1:**
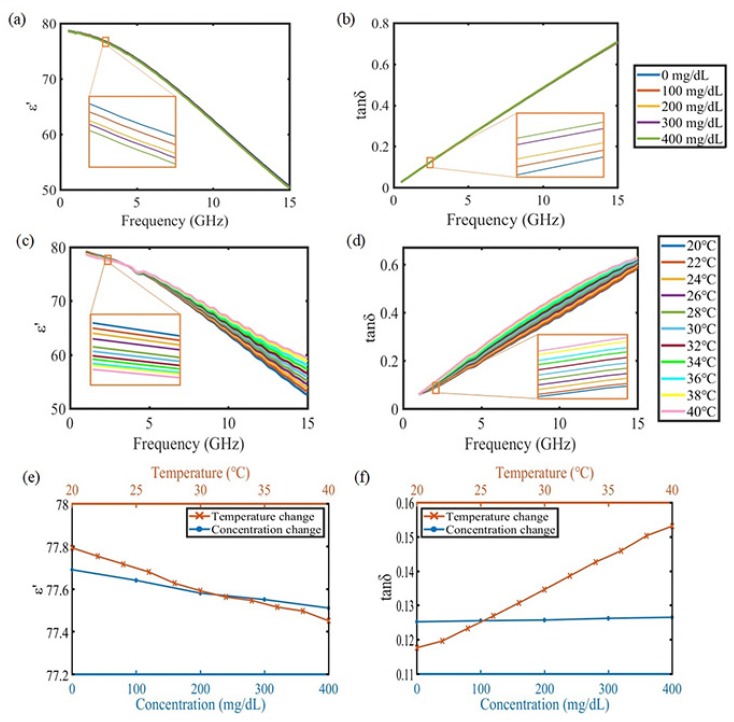
Electrical properties of glucose solution. (**a**) dielectric constant for various concentration at $25.5{\;}^{\circ }C$; (**b**) loss tangent for various concentration at $25.5{\;}^{\circ }C$; (**c**) dielectric constant of deionized water for various temperature; (**d**) loss tangent of DI water for various temperature; (**e**) change in dielectric constant at 2.9 GHz; (**f**) change in loss tangent at 2.9 GHz.

To demonstrate the effect of glucose concentration and ambient temperature on electrical properties, tests are conducted under various conditions. The real part of the complex relative permittivity ($\varepsilon^{\prime }$) and the loss tangent of the SUT at temperature of $25.5{\;}^{\circ }C$ are shown in Figure 1a,b. The dielectric constant decreases as the glucose concentration or frequency increase. On the other hand, the loss tangent increases with an increase in the glucose concentration or frequency. In another condition, the electrical properties of the sample (deionized water) when the temperature of the sample is increased from $20{\;}^{\circ }C$ to $40{\;}^{\circ }C$ with a step of $2{\;}^{\circ }C$ are shown in Figure 1c,d. The effect of increase in temperature and glucose concentration has a similar tendency to the electrical properties. Figure 1e,f indicate the change in the dielectric constant and loss tangent, respectively, with increasing the glucose concentration or temperature at a specific frequency (2.9 GHz). As shown in these results, it is essential to remove the influence of temperature change for accurate glucose concentration detection.

### 2.2. Description of the Proposed Sensor

The microwave resonator is a device that has frequency-selective characteristics because it resonates at a specific frequency, called resonant frequency, at which the stored energies in the frequency-dependent parasitic inductor and capacitor are equal. CSRR has been widely used to characterize the materials due to high sensitivity and its electromagnetic properties [32,33,34]. In this study, a CSRR, which is a type of microwave resonator, has been designed for non-invasive detection of glucose concentration. The CSRR is a device that the ground plane of microstrip line is etched in the form of double split-ring. Since the microstrip line is a typical transmission line and resonance occurs due to double split-ring composed of square-shaped loops with a gap, the CSRR has bandstop characteristics.

**Figure 2:**
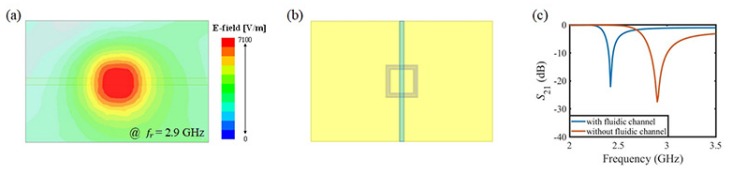
Simulation results of the proposed sensor. (**a**) electric field distribution at the resonant frequency (λ/15 above the substrate); (**b**) modelling of proposed sensor and fluidic channel as experimental condition; (**c**) transmission characteristic with and without the fluidic channel.

The electric field distribution of the CSRR at the resonant frequency is illustrated in Figure 2a. As can be inferred from this figure, an electromagnetic field called near-field is strongly confined within the double split-ring at the resonant frequency. When a material exists in the near-field region, the interaction between near-field and electrical properties of the material induces the variation in intrinsic characteristics of the resonator. Since the sensitivity in the measurement can be improved when a stronger near-field is formed, the center of the split-ring is set as a sensing region. To determine the resonant frequency of the proposed sensor, the experimental condition is simulated before fabricating the sensor. The fluidic channel on the sensing region of the proposed sensor is described in Figure 2b. The frequency response is obtained by the simulation using a three-dimensional full-wave electromagnetic solver. The simulation result is shown in Figure 2c. The resonant frequency of the proposed sensor shifted from 2.9 GHz to 2.42 GHz due to the higher permittivity of the DI water than the air. Consequently, the bare resonator is designed to resonate at 2.9 GHz in order to locate the resonant frequency in the ISM band (from 2.4 GHz to 2.45 GHz), when the fluidic channel passing over the resonator.

**Figure 3:**
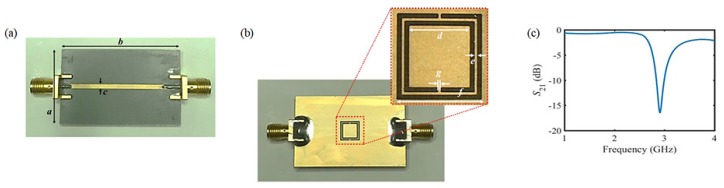
The fabricated sensor and its resonant characteristics. (**a**) signal line of the complementary split-ring resonator (CSRR); (**b**) ground plane of the CSRR and zoom-in of the resonant part; (**c**) measurement result of the transmission characteristic.

The proposed sensor is prepared as follows: a resonator is fabricated by standard photolithography and a chemical etching process. The signal line and ground plane are constructed with gold-plated copper with a thickness of 35 μm. The proposed sensor was fabricated on a Neltec NY9217(IM) substrate, which is 0.8 mm thickness with a dielectric constant (ε’) of 2.17 and loss tangent (tanδ) of 0.0008. The signal line and ground plane of the fabricated sensor with the dimensions given in Table 1 is shown in Figure 3a,b. The measured result of the transmission characteristics of the fabricated sensor is shown in Figure 3c. The sensor indicates a transmission coefficient of −17 dB at approximately 2.9 GHz.

### 2.3. Sensing Mechanism

**Figure 4:**
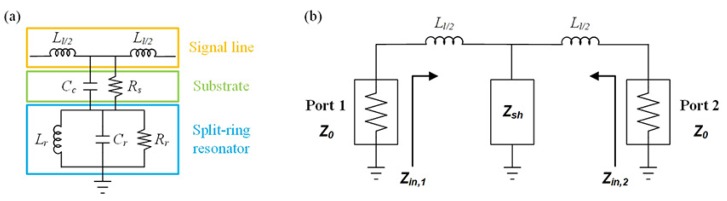
The equivalent circuit model of the proposed sensor. (**a**) complementary split-ring resonator; (**b**) simplified circuit model with port termination for transmission coefficient.

A straightforward way to analyze the planar microwave resonator is an equivalent circuit model. The equivalent circuit model is depicted in Figure 4a [35]. The signal line of the CSRR can be modeled as an inductor ($L_{\ell }$), and the electric coupling between the signal line and the ground plane can be modeled as a capacitor ($C_{c}$). The substrate loss is represented as a resistor ($R_{s}$). The loop, the gap, and the loss factor of the split-ring is equivalent to an inductor ($L_{r}$), a capacitor ($C_{r}$), and a resistor ($R_{r}$), respectively. To simplify the equivalent circuit model, the shunt branch of Figure 4a is modeled as one impedance block $Z_{sh}$.

The impedance of the shunt branch is given by:

(3) $$\begin{array}{c} Z_{\mathrm{sh}}=R_{\mathrm{sh}}+jX_{\mathrm{sh}}. \end{array}$$

The resistance ($R_{sh}$) and reactance ($X_{sh}$) of the shunt branch can be expressed as:

(4) $$\begin{array}{c} R_{\mathrm{sh}}=\frac{R_{s}}{1+\omega^{2}C_{c}^{2}R_{s}^{2}}+\frac{\omega^{2}L_{r}^{2}R_{r}}{{\left(R_{r}-\omega^{2}L_{r}C_{r}R_{r}\right)}^{2}+{\left(\omega L_{r}\right)}^{2}}, \end{array}$$

(5) $$\begin{array}{c} X_{\mathrm{sh}}=\frac{-\omega C_{c}R_{s}^{2}}{1+\omega^{2}C_{c}^{2}R_{s}^{2}}+\frac{\omega L_{r}R_{r}^{2}\left(1-\omega^{2}L_{r}C_{r}\right)}{{\left(R_{r}-\omega^{2}L_{r}C_{r}R_{r}\right)}^{2}+{\left(\omega L_{r}\right)}^{2}}, \end{array}$$

where ω is the angular frequency. To investigate the relationship between the lumped elements and transmission characteristics, the ports of the both ends are terminated as shown in Figure 4b. The transmission coefficient can be obtained by driving port 1 with an incident wave of voltage $V_{1}^{+}$ and measuring the outcoming wave amplitude at port 2,$V_{2}^{-}$, when the incident wave on port 2 ($V_{2}^{+}$) is set to zero by terminating the port. Then, the log scale of the transmission coefficient is defined as:

(6) $$\begin{array}{c} S_{21}=20\log_{10}{\left|\frac{V_{2}^{-}}{V_{1}^{+}}\right|}_{V_{2}^{+}=0}\;(\mathrm{dB}). \end{array}$$

$V_{2}^{-}$ can found by applying a voltage $V_{1}^{+}$ at port 1 and using voltage division twice as follows:

(7) $$\begin{array}{c} V_{2}^{-}=V_{1}^{+}\left(1+\Gamma_{1}\right)\left(1+\Gamma_{2}\right)\frac{\left(j\omega L_{\ell /2}+Z_{0}\right)∥Z_{\mathrm{sh}}}{j\omega L_{\ell /2}+\left[\left(j\omega L_{\ell /2}+Z_{0}\right)∥Z_{\mathrm{sh}}\right]}\frac{Z_{0}}{j\omega L_{\ell /2}+Z_{0}} \end{array}$$

(8) $$\begin{array}{c} =V_{1}^{+}\left(1+\frac{Z_{\mathrm{in},1}-Z_{0}}{Z_{\mathrm{in},1}+Z_{0}}\right)\left(1+\frac{Z_{0}-Z_{\mathrm{in},2}}{Z_{0}+Z_{\mathrm{in},2}}\right)\frac{\left(j\omega L_{\ell /2}+Z_{0}\right)∥Z_{\mathrm{sh}}}{j\omega L_{\ell /2}+\left[\left(j\omega L_{\ell /2}+Z_{0}\right)∥Z_{\mathrm{sh}}\right]}\frac{Z_{0}}{j\omega L_{\ell /2}+Z_{0}}, \end{array}$$

where $\Gamma_{1}$ and $\Gamma_{2}$ are reflection coefficient at the port 1 and port 2. Moreover, *Z_in_*_,1_ and *Z_in_*_,2_ are the input impedance seen looking toward the circuit at the port 1 and port 2. These are same values because of the structural symmetry, i.e.,

(9) $$\begin{array}{c} Z_{\mathrm{in}}=Z_{\mathrm{in},1}=Z_{\mathrm{in},2} \end{array}$$

(10) $$\begin{array}{c} =\left[\left(j\omega L_{\ell /2}+Z_{0}\right)∥Z_{\mathrm{sh}}\right]+j\omega L_{\ell /2} \end{array}$$

(11) $$\begin{array}{c} =\frac{-\omega^{2}L_{\ell /2}^{2}+Z_{0}Z_{\mathrm{sh}}+j\omega L_{\ell /2}\left(Z_{0}+2Z_{\mathrm{sh}}\right)}{Z_{0}+Z_{\mathrm{sh}}+j\omega L_{\ell /2}}. \end{array}$$

With Equations (6), (8) and (11), the transmission coefficient in log scale can be expressed in terms of lumped elements as follows:

(12) $$\begin{array}{c} S_{21}=20\log_{10}\left|\frac{4Z_{0}^{2}Z_{\mathrm{sh}}}{\left(j\omega L_{\ell /2}+Z_{0}+Z_{\mathrm{sh}}\right){\left(Z_{\mathrm{in}}+Z_{0}\right)}^{2}}\right|\;(\mathrm{dB}). \end{array}$$

Therefore, the increase in the concentration of the glucose solution leads to an increase in shunt impedance and transmission coefficient. Based on this principle, the concentration of the glucose solution can be estimated from the variation in transmission coefficient of the resonator as a sensing parameter.

## 3. Results and Discussion

### 3.1. Experimental Result under Stable Temperature Condition

**Figure 5:**
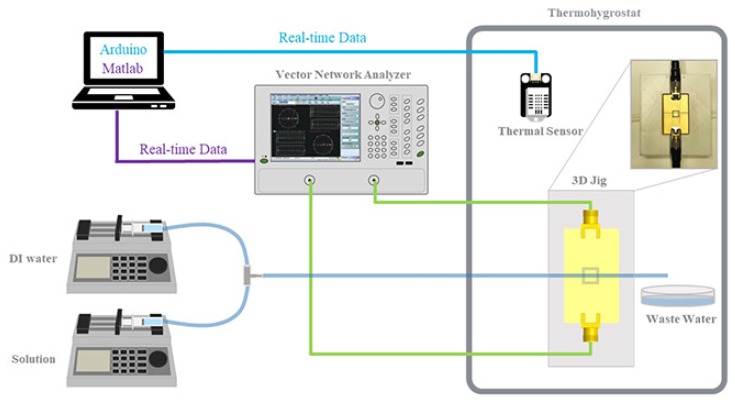
Measurement setup for temperature control and testing the performance of the proposed sensor.

The proposed glucose sensor is fabricated as described in Section 2.2, and the SMA connectors are soldered at the input and output of the signal line to connect with the measurement equipment, as depicted in Figure 3a,b. The measurement setup is illustrated in Figure 5. The fabricated microwave sensor is placed in a thermohygrostat to provide stable temperature conditions. The ports of the vector network analyzer (Agilent Technologies, E5071B) are connected to the two soldered connectors. The illustrated fluidic system consists of two syringe pumps that contain DI water and glucose solution of a concentration of 400 mg/dL with one outlet from the mixer. The glucose concentration of the outlet is varied from 0 mg/dL to 400 mg/dL with a step of 100 mg/dL every 2 min by controlling the flow rates of the two syringe pumps. The outlet mimics a blood vessel and is fixed using a 3D-printed jig, which enables it to pass over the center of the split-ring where the electric field is strongest, as shown in the inset of Figure 5. In addition, the temperature is monitored in real time by placing a commercial thermal sensor in the thermohygrostat. Last, the vector network analyzer and computer are connected using a GPIB cable, and the measurement data are acquired every second.

**Figure 6:**
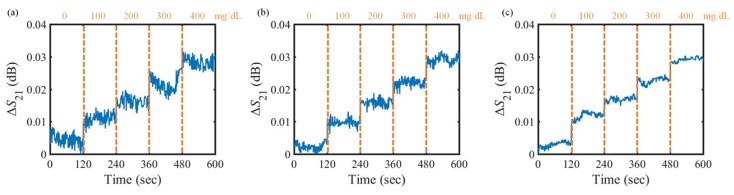
Measured result of the transmission coefficient variation under stable temperature condition. (**a**) $20{\;}^{\circ }C$; (**b**) $30{\;}^{\circ }C$; (**c**) $40{\;}^{\circ }C$.

The maintained temperature is set to $20{\;}^{\circ }C$, $30{\;}^{\circ }C$, $40{\;}^{\circ }C$. Figure 6 shows the variation in the transmission coefficient of the proposed glucose sensor when the concentration of the glucose solution is changed every 2 min at a given temperature. To quantitatively analyze the performance of the proposed sensor, a parameter is defined as follows:

(13) $$\begin{array}{c} \Delta S_{21}=S_{21,\mathrm{concentration}}-S_{21,\mathrm{DI}{\mathrm{water}}_{\min}}\;(\mathrm{dB}), \end{array}$$

where $S_{21,concentration}$ is the transmission coefficient at the resonant frequency in each concentration of the glucose solution, whereas $S_{21,DIwater_{min}}$ represents the minimum value of the transmission coefficient at the resonant frequency when the DI water flows through the fluidic channel. As shown in Figure 6, the proposed sensor exhibits an approximately 0.03 dB variation in the transmission coefficient when the concentration of the glucose solution increases from 0 mg/dL to 400 mg/dL.

### 3.2. Experimental Result under Varying Temperature Conditions

**Figure 7:**
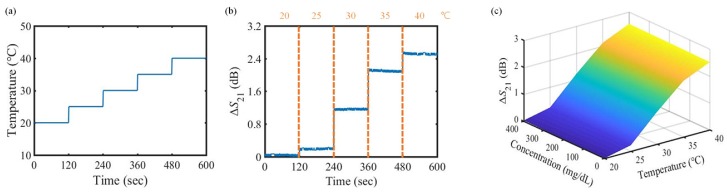
Relationship between the temperature and the transmission coefficient of the proposed sensor. (**a**) variation in temperature increases by $5{\;}^{\circ }C$ from $20{\;}^{\circ }C$ to $40{\;}^{\circ }C$ every 2 min; (**b**) variation in transmission coefficient under varying temperature condition when the DI water flows through the fluidic channel; and (**c**) variation in transmission coefficient for the other concentrations of the glucose solution under varying temperature condition.

Note that the electrical property of the liquid is influenced by the temperature. To guarantee the reliability and repeatability of the measured data, the performance of the proposed sensor as a function of temperature should be tested, and the effect of the temperature should be compensated. For the temperature test, the temperature inside the thermohygrostat of Figure 5 is varied from $20{\;}^{\circ }C$ to $40{\;}^{\circ }C$ at intervals of $5{\;}^{\circ }C$ to handle not only the room temperature (around $20{\;}^{\circ }C$) but also the body temperature (around $36{\;}^{\circ }C$), as shown in Figure 7a. The temperature transition time is disregarded. Figure 7b shows the variation in the transmission coefficient when the DI water flows through the fluidic channel under the temperature condition given in Figure 7a. Despite being carried out in the same concentration (DI water), the variation in the transmission coefficient due to a temperature change of $20{\;}^{\circ }C$ is greater than that due to concentration change of 400 mg/dL (Section 3.1). The results of varying the temperature for the other concentrations is shown in Figure 7c. As can be inferred from this figure, the effect of temperature change is more significant than the effect of glucose concentration change on the variation in the transmission coefficient of the sensor. Therefore, the temperature correction is essential for accurate detection of the glucose concentration.

For the temperature correction, the fitting function is derived from the relationship between the temperature and variation in transmission coefficient for DI water, and then applied to the case in which the glucose concentration and temperature are simultaneously changed. Consequently, a temperature correction function is obtained as follows:

(14) $$\begin{array}{c} \Delta S_{21,\mathrm{temp}}=-0.00081T^{3}+0.0735T^{2}-2.027T+17.57\;(\mathrm{dB}), \end{array}$$

where ΔS21,temp indicates the variation in the transmission coefficient as a function of temperature *T* in degrees Celsius (${\;}^{\circ }C$).

**Figure 8:**
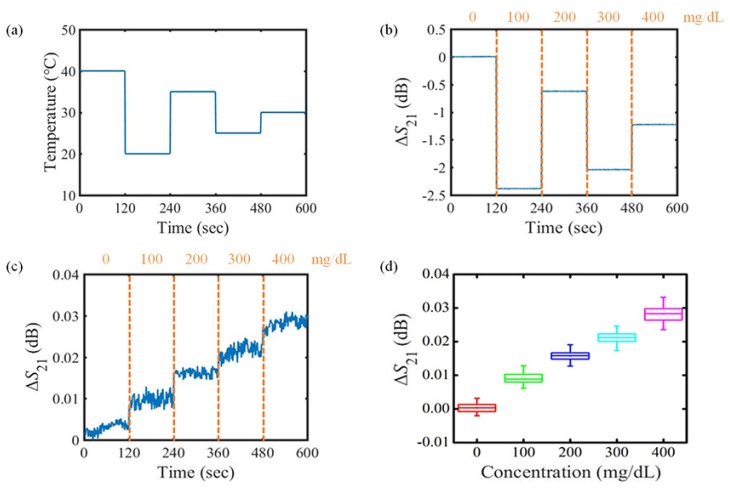
Measured results of variation in the transmission coefficient for varying temperature condition. (**a**) temperature set arbitrarily to address both room temperature ($20{\;}^{\circ }C$) and body temperature ($36{\;}^{\circ }C$); (**b**) variation in transmission coefficient before the temperature correction; (**c**) variation in transmission coefficient after the temperature correction; (**d**) statistical result for variation in the transmission coefficient after temperature correction obtained from repeated experiment.

To validate the derived temperature correction function, the ambient temperature is arbitrarily changed and Equation (14) is applied. Thereafter, the performance correction of the proposed sensor for different concentrations is observed. As shown in Figure 8a, the temperature is adjusted to $40{\;}^{\circ }C$, $20{\;}^{\circ }C$, $35{\;}^{\circ }C$, $25{\;}^{\circ }C$, $30{\;}^{\circ }C,$ respectively, as the concentration of the glucose solution is monotonically increased from 0 mg/dL with a 100 mg/dL step. Since the effect of the temperature change is more dominant than that of the concentration change, the variation in the transmission coefficient of the sensor follows the temperature variation as described in Figure 8b. In order to eliminate the undesired effect due to the temperature, Equation (14) for the DI water is applied to Figure 8b, and the final result is plotted in Figure 8c. After temperature correction, as the concentration of the glucose solution increases from 0 mg/dL to 400 mg/dL, the transmission coefficient of the proposed sensor showed a variation of 0.03 dB, the same as in the case of a stable temperature condition. To demonstrate the reproducibility of the proposed sensor, the temperature is randomly changed at the same time as the concentration of the glucose solution that flows through the fluidic channel is increased by 100 mg/dL every two minutes, and the transmission coefficient of the sensor is obtained by eight iterations. In this order, by applying Equation (14) to each data, the undesired effect of the temperature is eliminated. The statistical result of repeated measurement is plotted in Figure 8d. In the box plot, the bounds of the boxes, the bars outside the boxes, the lines inside the boxes, and the squares inside the boxes represent the 25% and 75% of the data distribution, the minimum and maximum values excluding outliers, the median of the data, and the mean value of the data, respectively. For the repeated measurement results shown in Figure 8d, the bound values (25%, 75%) of the boxes and mean value for each concentration are summarized in Table 2.

This result indicates that each glucose concentration can be clearly distinguished by the transmission coefficient, even in repeated measurements. As a result, even if the atmosphere temperature or body temperature changes, the proposed sensor can detect the normal range of blood glucose concentration as well as the judgement of hypoglycemia or hyperglycemia state.

## 4. Conclusions

In this study, a temperature-corrected non-invasive glucose sensor has been developed. The proposed sensor is based on a CSRR and can detect the concentration of glucose solution from 0 mg/dL to 400 mg/dL with a fluidic system. The proposed glucose sensor has been tested in two conditions: stable temperature and varying temperature conditions. Under the condition in which the temperature changes, the effect of temperature is eliminated by applying a derived correction function from the relationship between the temperature and the transmission coefficient variation of the proposed sensor for the DI water. In the stable temperature condition, the performance of the proposed glucose sensor showed that the transmission coefficient varied by 0.03 dB when the concentration of the glucose solution increased from 0 mg/dL to 400 mg/dL. Likewise, for the temperature varying condition, the variation in the transmission coefficient is 0.03 dB, which is similar to the stable temperature condition, excluding the undesired effect of the temperature. These results indicate that the proposed glucose sensor has a possibility of non-invasive and continuous monitoring of the glucose concentration at any reasonable temperature range. In this paper, the fluidic channel that mimics the human blood vessel is used. The most accurate way to detect the blood glucose concentration is to use venous blood plasma. Human tissue consists of skin, fat, and muscle layers in order from outside. In addition, the muscle is a component that is electrically lossy compared to skin and fat layers. Therefore, an electromagnetic wave can penetrate deeper into the body part where there is less lossy muscle. For these reasons, the proposed sensor can be utilized to the patient by attaching the proposed sensor near the subclavian vein around the collarbone. Future work will focus on the selectivity. Among the many constituents of blood, the concentration of sodium chloride and glucose is changed dramatically before and after the meal. Thus, the discrimination between sodium chloride and glucose will be studied.

## Figures and Tables

**Table 1:** Dimensions of the proposed sensor.

Parameter	a	b	c	d	e	f	g
mm	26	40	2.4	5.02	0.22	7	0.22

**Table 2:** Summary of the statistical distribution.

Concentration (mg/dL)	Δ$S_{21}$ (×10${}^{-3}$ dB)		
	25%	Mean	75%
0	1.2	2.3	3.3
100	8.0	9.1	10.3
200	14.8	15.7	16.7
300	20.0	21.2	22.3
400	26.4	28.2	29.8
